# P-598. Attitudes toward a potential Norovirus vaccine among patients with medically-attended acute gastroenteritis in the US

**DOI:** 10.1093/ofid/ofae631.796

**Published:** 2025-01-29

**Authors:** Holly C Groom, Matthew T Slaughter, Judy L Donald, Emma Viscidi, John F Dickerson, Maureen O’Keeffe-Rosetti, Mark A Schmidt, Laura H Hendrix, Katherine B Carlson

**Affiliations:** Kaiser Permanente Center for Health Research, Portland, Oregon; Kaiser Permanente Northwest Center for Health Research, Portland, Oregon; Kaiser Permanente Center for Health Research, Portland, Oregon; Moderna Therapeutics, Cambridge, Massachusetts; Kaiser Permanente Center for Health Research, Portland, Oregon; Kaiser Permanente Center for Health Research, Portland, Oregon; Center for Health Research, Kaiser Permanente Northwest, Portland, Oregon; Moderna Therapeutics Ltd., Greensboro, Massachusetts; Moderna, Cambridge, Massachusetts

## Abstract

**Background:**

Clinical trials for norovirus (NoV) vaccines are underway. Prior research has found generally positive attitudes toward a potential NoV vaccine, but there is limited understanding of how the COVID-19 pandemic may have impacted attitudes toward novel vaccines.

**Methods:**

We identified medically-attended acute gastroenteritis (MAAGE) encounters among Kaiser Permanente Northwest members of all ages based on ICD-10 diagnostic codes, occurring from 11/17/2023-4/18/2024. Participants were asked to complete surveys which included questions about their level of agreement with the need for and willingness to get a potential NoV, as well as whether the COVID-19 pandemic impacted attitudes about vaccines.

**Figure 2.**
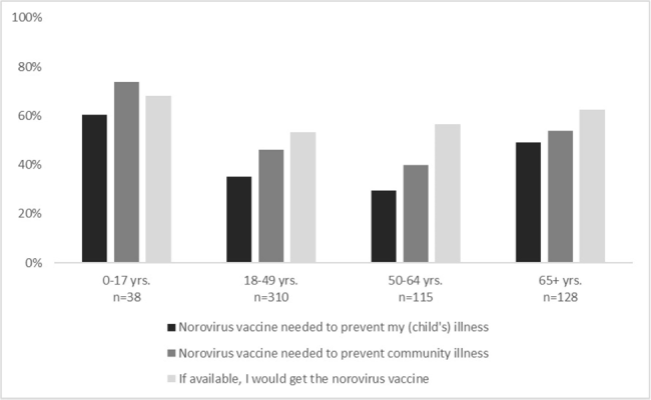
How has the COVID-19 pandemic changed attitudes about vaccines? Distribution of responses, by age group, November 2023-April 2024.

**Results:**

Of 8,363 adults and 666 parents/guardians of children with MAAGE who were invited to participate, 553 (6.6%) adults and 38 (5.8%) guardians consented and completed surveys. Among adults, median age was 46 (range 18-90) years; most were female (76%) and of white race (84%). Among children of parents/guardians, median age was 4 (range 0-17) years; 58% were female and 89% were white.

Overall, 37% of adult MAAGE patients agreed that a NoV vaccine was needed to prevent illness for themselves and 47% agreed that a vaccine was needed to protect the community; 56% agreed that they would get a NoV vaccine if available. When stratified by age group (18-49 yrs., 50-64 yrs., 65+ yrs.), the oldest respondents had the most favorable responses, with 63% of adults 65+ years indicating that they would get a potential vaccine (Fig 1).

Among guardians, 61% agreed a vaccine was needed to prevent their child from getting ill and 74% agreed the community was in need of a NoV vaccine. Overall, 68% agreed they would vaccinate their child. When asked how the COVID-19 pandemic had impacted attitudes about vaccines, most respondents indicated there was either no change (37-42%) or they had increasingly positive attitudes (26-41%); a minority (6-16%) reported more negative attitudes (Fig 2).

**Figure:**
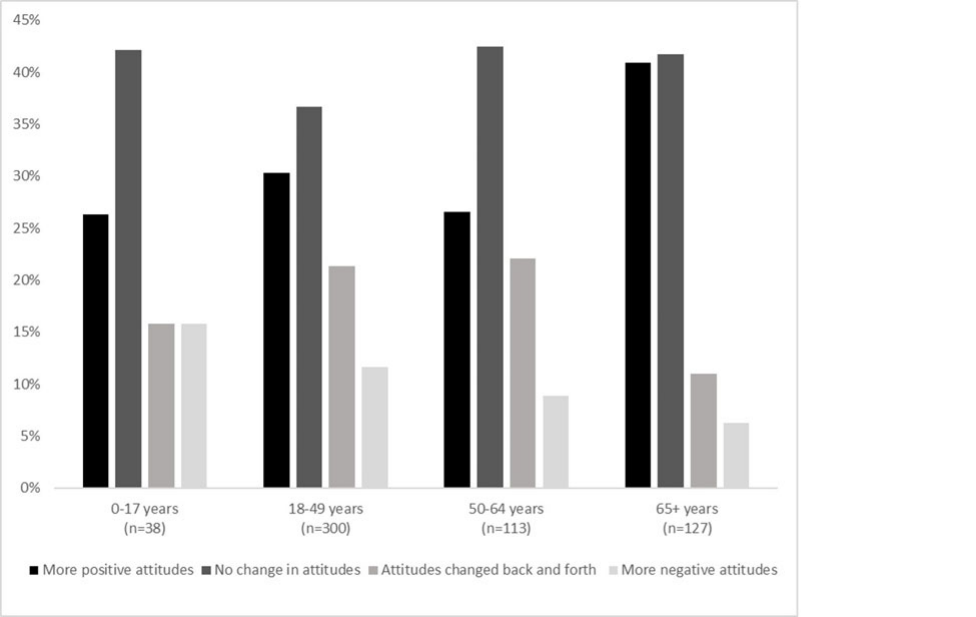

**Conclusion:**

In this sample of patients with recent MAAGE events, over 60% of adults 65+ and child guardians expressed willingness to get a NoV vaccine for themselves or their child. More

respondents reported a positive shift in comparison to a negative shift in attitude towards vaccines following the COVID-19 pandemic.

**Disclosures:**

**Holly C. Groom, MPH**, Hillevax: Grant/Research Support|Moderna: Grant/Research Support **Matthew T. Slaughter, MS**, Moderna: Grant/Research Support **Judy L. Donald, MA**, HilleVax: Grant/Research Support|Janssen: Grant/Research Support|Moderna: Grant/Research Support|Pfizer: Grant/Research Support|Vir Biotechnology: Grant/Research Support **Emma Viscidi, PhD, MHS**, Moderna: Stocks/Bonds (Public Company) **John F. Dickerson, PhD**, Janssesn: Grant/Research Support|Moderna: Grant/Research Support|Novartis: Grant/Research Support|Pfizer: Grant/Research Support **Maureen O'Keeffe-Rosetti, MS**, Hillevax: Grant/Research Support|Janssen: Grant/Research Support|Moderna: Grant/Research Support|Pfizer: Grant/Research Support **Mark A. Schmidt, PhD, MPH**, HilleVax: Grant/Research Support|Janssen: Grant/Research Support|Moderna: Grant/Research Support|Pfizer: Grant/Research Support|Vir Biotechnology: Grant/Research Support **Laura H. Hendrix, MS**, Moderna Therapeutics Ltd.: Stocks/Bonds (Private Company) **Katherine B. Carlson, PhD, MPH**, Moderna: Stocks/Bonds (Private Company)

